# Lactate and Lactylation in AKI‐to‐CKD: Epigenetic Regulation and Therapeutic Opportunities

**DOI:** 10.1111/cpr.70034

**Published:** 2025-04-10

**Authors:** Yi Hou, Dongwei Liu, Zuishuang Guo, Cien Wei, Fengyu Cao, Yue Xu, Qi Feng, Fengxun Liu

**Affiliations:** ^1^ Department of Nephrology The First Affiliated Hospital of Zhengzhou University Zhengzhou Henan Province China; ^2^ Traditional Chinese Medicine Integrated Department of Nephrology The First Affiliated Hospital of Zhengzhou University Zhengzhou Henan Province China; ^3^ Research Institute of Nephrology, Zhengzhou University Zhengzhou Henan Province China; ^4^ Henan Province Research Center for Kidney Disease The First Affiliated Hospital of Zhengzhou University Zhengzhou Henan Province China; ^5^ Key Laboratory of Precision Diagnosis and Treatment for Chronic Kidney Disease in Henan Province The First Affiliated Hospital of Zhengzhou University Zhengzhou Henan Province China; ^6^ Tianjian Laboratory of Advanced Biomedical Sciences, Academy of Medical Sciences, Zhengzhou University Zhengzhou Henan Province China; ^7^ Innovation Center of Basic Research for Metabolic‐Associated Fatty Liver Disease, Ministry of Education of China Zhengzhou Henan Province China

**Keywords:** acute kidney injury (AKI), chronic kidney disease (CKD), lactate, lactylation

## Abstract

Lactate is not only a byproduct of glycolysis, but is also considered an energy source, gluconeogenic precursor, signalling molecule and protein modifier during the process of cellular metabolism. The discovery of lactylation reveals the multifaceted functions of lactate in cellular metabolism and opens new avenues for lactate‐related research. Both lactate and lactylation have been implicated in regulating numerous biological processes, including tumour progression, ischemic–hypoxic injury, neurodevelopment and immune‐related inflammation. The kidney plays a crucial role in regulating lactate metabolism, influencing lactate levels while also being regulated by lactate. Previous studies have demonstrated the importance of lactate in the pathogenesis of acute kidney injury (AKI) and chronic kidney disease (CKD). This review explores the role of lactate and lactylation in these diseases, comparing the function and metabolic mechanisms of lactate in normal and diseased kidneys from the perspective of lactylation. The key regulatory roles of lactylation in different organs, multiple systems, various pathological states and underlying mechanisms in AKI‐to‐CKD progression are summarised. Moreover, potential therapeutic targets and future research directions for lactate and lactylation across multiple kidney diseases are identified.

## Graphical abstract

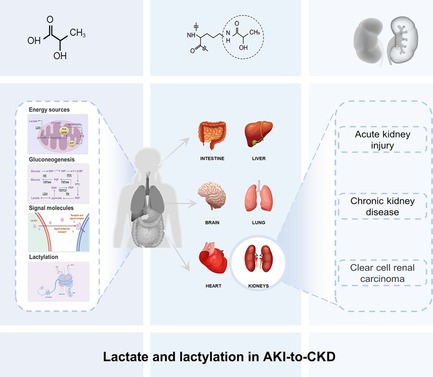

**Abbreviations**
A/I: blation and infarction
AA: aristolochic acid
AARS1/2: aminoacyl‐tRNA synthetase 1/2
ACC: acetyl‐CoA carboxylase
ACSF2: acyl‐CoA synthetase family member 2
AD: Alzheimer's disease
AKI: acute kidney injury
ALI: acute liver injury
ARB: angiotensin II receptor blocker
ATP: adenosine triphosphate
BCAP: B‐cell adapter for PI3K
BUB1B: BUB1 mitotic checkpoint serine/threonine kinase B
Caspase‐1: cysteine‐aspartic protease 1
ccRCC: clear cell renal carcinoma
CKD: chronic kidney disease
CobB: cobalamin‐dependent protein
CRC: colorectal cancer
DN: diabetes nephropathy
Drp1: dynamin‐related protein 1
EMT: epithelial—mesenchymal transition
ENO1: enolase 1
FA: folic acid
Fis1: mitochondrial fission 1 protein
GBM: glioblastoma
GFR: glomerular filtration rate
GLIS1: Gli‐like transcription factor 1
Glis1: GLIS family zinc finger 1
Gln: glutamate
GLUT1: glucose transporter 1
GPRs: G protein‐coupled receptors
GSDMD: gasdermin D
H/R: hypoxia/reoxygenation
H3K14: histone H3 lysine 14
H3K18la: histone H3 lysine 18 lactylation
H3K9: histone H3 lysine 9
H4K12la: histone H4 lysine 12 lactylation
H4K5: histone H4 lysine 5
HATs: histone acetyltransferases
HCC: hepatocellular carcinoma
HDACs: histone deacetylases
HIF‐1α: hypoxia‐inducible factor 1‐alpha
HK‐2 cell: human kidney 2 cell
HK2: hexokinase 2
HPLC: high‐performance liquid chromatography
I/R: ischemia–reperfusion
IDH3β: isocitrate dehydrogenase 3 beta
KLF5: Krüppel‐like factor 5
L/A: lactate/albumin
LDH: lactate dehydrogenase
LGSH: lactylglutathione
LPS: lipopolysaccharide
MCTs: monocarboxylate transporters
MDSCs: myeloid‐derived suppressor cells
MI: myocardial infarction
MS: mass spectrometry
mTORC1: mechanistic target of rapamycin complex 1
NAD‐to‐NADH: nicotinamide adenine dinucleotide‐to‐reduced nicotinamide adenine dinucleotide
NLRP3: NOD‐like receptor family pyrin domain‐containing protein 3
NSUN2: NOP2/Sun RNA methyltransferase 2
NUSAP1: nucleolar spindle‐associated protein 1
PAX6: paired box 6
PCa: prostate cancer
PD‐1: programmed cell death protein 1
PDGFRβ: platelet‐derived growth factor receptor β
PDH: pyruvate dehydrogenase
PDHA1: pyruvate dehydrogenase E1 component subunit alpha
PD‐L1: programmed cell death ligand 1
PFKFB3: 6‐phosphofructo‐2‐kinase/fructose‐2,6‐biphosphatase 3
PGC1‐α: peroxisome proliferator‐activated receptor γ coactivator‐1α
PKD: polycystic kidney disease
PKM2: pyruvate kinase M2
PTCs: proximal tubule cells
PTECs: proximal tubular epithelial cells
PTMs: posttranslational modifications
RhoA: Ras homologue gene family member A
ROCK: RhoA/Rho‐associated protein kinase
ROS: reactive oxygen species
RRT: renal replacement therapy
SAKI: sepsis‐associated acute kidney injury
SIRT: sirtuin
SLC16: solute carrier family 16
TAMs: tumour‐associated macrophages
TCA cycle: tricarboxylic acid cycle
TECs: tubule epithelial cells
TIP60: Tat‐interacting protein 60
TME: tumour microenvironment
TTK: threonine tyrosine kinase
UC: ulcerative colitis
UUO: unilateral ureteral obstruction
VHL: Von Hippel–Lindau

## 1 Introduction

The multifaceted roles of lactate, including ‘energy sources’, ‘gluconeogenic precursors’ and ‘signal transduction molecules’, exert significant regulatory effects across different organs, diseases and systems. Lactate has been widely used as a prognostic indicator for critically ill patients [1]. However, the precise regulatory mechanisms involved in human diseases remain unclear. In 2019, Yingming Zhao's group discovered the epigenetic regulatory function of lactate and introduced the concept of lactylation [2]. This discovery pioneered a novel field of research into protein posttranslational modifications (PTMs) while elucidating the potential mechanisms by which lactate influences metabolism and gene expression. These findings provide new directions for investigating the underlying mechanisms of metabolic abnormalities in various diseases, including immune‐related inflammation, tumour proliferation, hypoxic injury and neurodevelopment. Currently, this field is still in its infancy, which prompted us to reevaluate previous lactate‐related studies from the perspective of protein modifications and explore potential research directions.

Lactate exists in two primary forms in the body: l‐lactate and d‐lactate. Generally, l‐lactate is the predominant form, whereas d‐lactate is a metabolite produced by intestinal bacteria [3]. Under normal physiological conditions, lactate serves as an essential fuel for the tricarboxylic acid (TCA) cycle, providing energy when blood glucose is insufficient [4, 5]. Additionally, lactate regulates the cellular redox balance and influences fatty acid metabolism [6, 7]. However, under pathological conditions, the regulatory role of lactate becomes more complex. Lactate involves numerous pathological processes, including inflammation, memory formation, neuroprotection, wound healing, ischaemic–hypoxic injury, tumour growth and metastasis [3, 8]. Moreover, it is implicated in diseases characterised by metabolic dysregulation.

Although lactate metabolism is intricate in all organs, the kidneys exhibit unique characteristics. Similar to the liver, the kidneys can produce lactate through glycolysis and break it down through gluconeogenesis. Additionally, the kidneys can excrete small amounts of lactate. Glycolysis converts glucose into pyruvate, which is subsequently transformed into lactate via lactate dehydrogenase (LDH) instead of entering the mitochondria for the TCA cycle [9]. Before the discovery of lactylation, it was widely believed that renal lactate primarily entered the TCA cycle or was reutilised as an energy source through gluconeogenesis to support metabolic functions [10, 11]. The complexity of lactate metabolism in the kidneys is further highlighted by the fact that this process is regulated by various hormones and physiological stimuli and varies between the renal cortex and medulla. The glucose**–**lactate cycle between the cortex and medulla facilitates substance transport in the kidneys through the interconversion of glucose and lactate. Despite the established importance of lactate metabolism in the kidneys, the specific regulatory mechanisms underlying this process remain poorly understood under different physiological and pathological conditions.

Lactate plays a significant role in the pathogenesis of acute kidney injury (AKI) and its progression to chronic kidney disease (CKD). Metabolic reprogramming, inflammation and microcirculatory dysfunction are key mechanisms in the AKI pathogenesis, with impaired gluconeogenesis being a key metabolic feature [12]. Elevated lactate levels are an independent risk factor for AKI and are positively correlated with increased patient mortality [13]. Consequently, lactate has emerged as an important clinical biomarker for assessing AKI progression. In CKD with various aetiologies, tubular epithelial cells undergo metabolic alterations, leading to lactate accumulation, which is closely associated with impaired glucose metabolism and renal fibrosis development. Therapeutically, inhibiting lactate production or enhancing its clearance could mitigate CKD progression [14]. Further investigation is needed to elucidate the role of lactate in the AKI‐to‐CKD progression.

Yingming Zhao's group initially demonstrated the occurrence of histone lysine lactylation by high‐performance liquid chromatography–tandem mass spectrometry (HPLC‐MS/MS). They reported that lactylation promotes the transition of macrophages from an inflammatory (M1) phenotype to a homeostatic (M2) phenotype during the later stages of polarisation [2]. Subsequent studies have identified several key enzymes and regulatory factors involved in histone lactylation and the occurrence of lactylation in nonhistone proteins. Although research on lactylation is still in its infancy, with limited literature available, it has already been demonstrated to exert broad regulatory effects on various pathophysiological processes. This finding is consistent with the critical role of lactate metabolism in numerous diseases. Given the significance of metabolic abnormalities in the AKI‐to‐CKD pathogenesis, it is warranted to investigate lactylation's involvement in disease progression and its effects on these mechanisms.

Currently, research on lactylation in kidney diseases is limited; however, existing studies have revealed that lactylation in AKI can affect mitochondrial function and inflammatory pathways. Elevated lactylation levels induce apoptosis and inflammation through mitochondrial fission protein 1 (Fis1) [15] and the Ras homologue gene family member A (RhoA)/RhoA/Rho‐associated protein kinase (ROCK) signalling pathway [16], thereby exacerbating disease progression. In CKD, lactylation regulates key glycolytic enzymes and the NF‐κB signalling pathway, promoting renal inflammation and fibrosis [17]. The elevated panlactylation level in diabetic nephropathy (DN) can influence mitochondrial function and metabolism in the kidneys, interfere with the cell cycle‐related gene expression and contribute to the DN progression [18, 19].

Reviewing previous studies on lactate in the progression of AKI to CKD alongside current research on lactylation in various contexts, including tumours, neurodevelopmental disorders and inflammatory diseases, may reveal promising research targets. Currently, available evidence remains insufficient to draw definitive conclusions. This review summarises the recently emerging lactate roles and their pathophysiological functions, grounded in the conceptual framework of lactylation. This review also highlights the pivotal role of the kidneys in lactate metabolism and associated metabolic processes. Finally, previous studies on lactate in patients with AKI to CKD are discussed and current findings concerning lactylation are summarised, aiming to acquire a more precise understanding of the correlation between the kidneys and lactate/lactylation. This review aims to identify potential research targets and provide valuable insights for further studies on lactate and lactylation in the AKI to CKD progression.

## 2 History of Studies on Lactate

**FIGURE 1:**
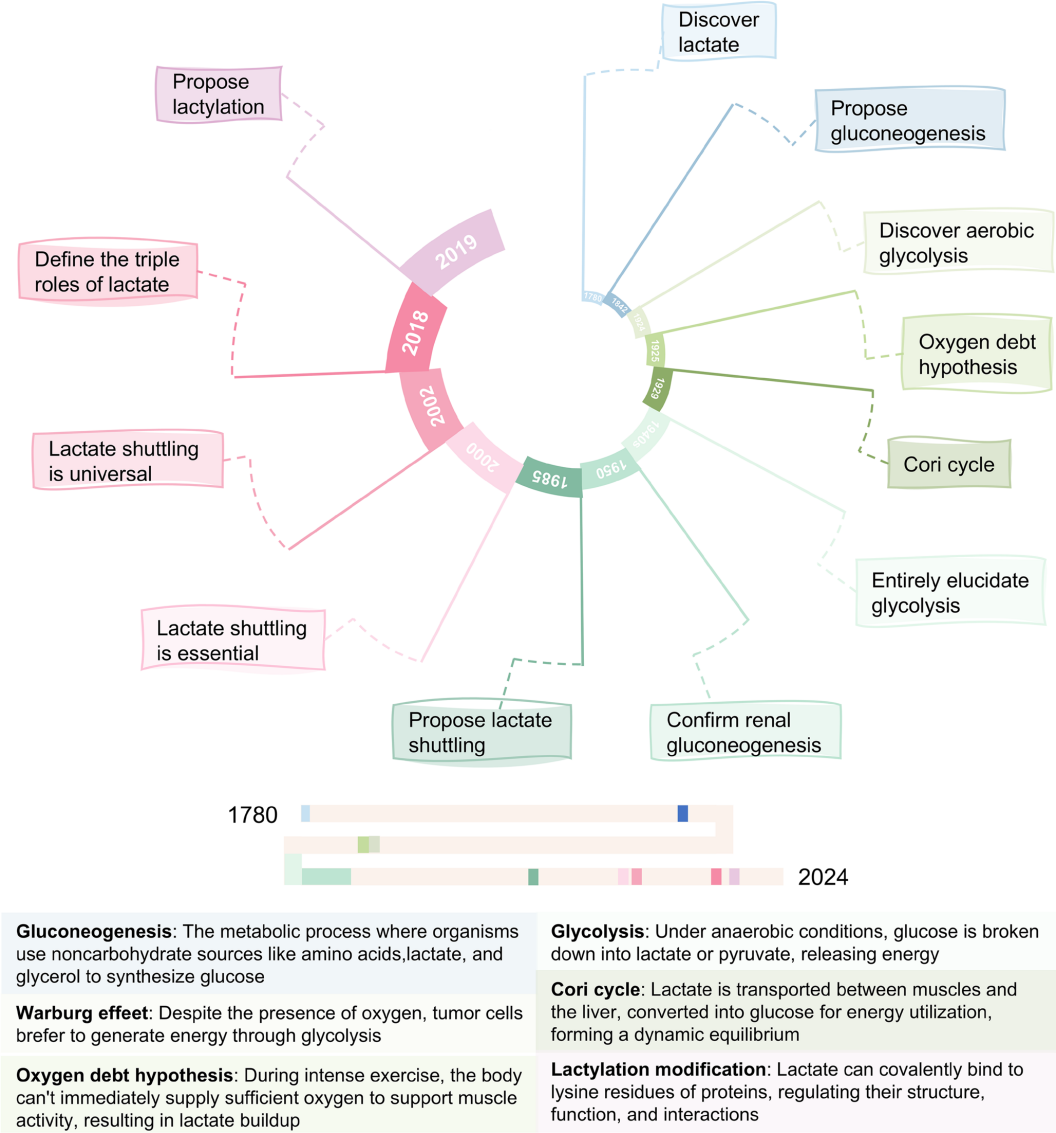
The timeline of the key discoveries related to advances in lactate research The key milestones in lactate‐related research are concisely summarised and highlighted in this timeline. Definitions of essential concepts are provided at the bottom of the figure. This timeline facilitates a deeper understanding of the critical role of lactate by tracing its research progress.

Lactate was first discovered over two centuries ago (Figure 1). Initially, research on lactate progressed slowly and scientists regarded it merely as a metabolic waste product generated under hypoxic conditions. With the introduction of the Warburg effect, the Cori cycle and the lactate shuttle, the molecular structure and production process of lactate have been clarified [1, 20, 21, 22]. Additionally, the three primary roles of lactate have been defined: Energy sources, gluconeogenic precursors and signalling molecules [23]. When investigating the role of lactate in signal transduction, Yingming Zhao's group in 2019 discovered that lactate could covalently bind to lysine residues on proteins, thereby regulating protein structure, function and interactions, a process known as lactylation [2].

## 3 The Functions of Lactate

### 3.1 Energy Regulation

Lactate is pivotal in glucose metabolism, serves as a key fuel for the TCA cycle and contributes to energy production. The lactate metabolic process can generate adenosine triphosphate (ATP) and promote glucose metabolism, thereby producing additional ATP. It can also inhibit glycolysis through product feedback pathways, stimulate oxidative phosphorylation and help maintain the relative stability of blood glucose and energy levels in the body [4, 5]. Additionally, lactate facilitates the maintenance of redox balance and tissue integrity, as well as the entire organism. High lactate concentrations increase the nicotinamide adenine dinucleotide‐to‐reduced nicotinamide adenine dinucleotide (NAD‐to‐NADH) ratio, resulting in excessive production of reactive oxygen species by mitochondria, which causes oxidative damage, accelerate cellular senescence and cause severe, irreversible damage to cells [6, 7]. Lactate can also increase the intracellular acetyl‐CoA level, enhance fatty acid synthase activity, inhibit fatty acid oxidation and promote fat storage [24, 25, 26]. In summary, lactate conversion plays a crucial role in energy metabolism.

### 3.2 Inflammation

The regulation of inflammation by lactate is complex and involves contradictory proinflammatory or anti‐inflammatory effects. During acute inflammation, lactate typically inhibits inflammation. It can suppress the production of proinflammatory factors, prevent mast cell degranulation and delay the upregulation of inflammation‐related genes in lipopolysaccharide (LPS)‐stimulated monocytes. Additionally, lactate can reduce the activation of the NF‐κB signalling pathway, contributing to immunosuppression in sepsis [27, 28, 29]. However, chronic inflammation is characterised by lactate‐driven immune dysfunction in T cells, inhibited T cell migration, increased production of inflammatory cytokines and prolonged inflammation. Furthermore, lactate stimulates the transition of macrophages to the M2 phenotype, contributing to fibrosis in various chronic diseases [30]. Clinically, lactate levels serve as important indicators for assessing the efficacy and prognosis of inflammation‐related diseases. Given that inflammation is involved in acute and chronic pathological conditions across nearly all organs, lactate plays a subtle yet significant role in these processes.

### 3.3 Tumours and Immunosuppression

As a hallmark of tumour cells characterised by abnormal metabolism, lactate levels in the tumour microenvironment can fluctuate between exceptionally high and low levels [31]. Lactate primarily serves as a vital energy source to meet the high proliferation demands of tumours [32]. Furthermore, a high‐lactate environment selectively favours the survival of more resilient tumour cells, promotes immune evasion and forms an immunosuppressive environment that supports tumour growth and survival [8, 33, 34]. The effects of lactate on immune cells are highly cell‐type specific. Lactate inhibits the functions of CD8^+^ T cells, natural killer T cells, macrophages and dendritic cells while promoting the immunosuppressive functions of myeloid‐derived suppressor cells and regulatory T cells [1, 3, 35]. Furthermore, lactate and its signalling pathway, which is activated by G protein‐coupled receptor 81 (GPR81), contribute to multiple aspects of tumour progression, including cell proliferation, invasion, angiogenesis, immune tolerance and immune cell evasion [10]. The immune evasion and metabolic reprogramming of tumour cells are closely linked by lactate molecules, which are unique and critically important in cancer research.

### 3.4 Cell Injury

Lactate, an indicator of insufficient tissue perfusion, is closely associated with poor prognosis in patients with ischemic–hypoxic injury [36, 37]. It exerts different regulatory effects on various types of cellular injury. For instance, in ulcerative colitis, elevated macrophage lactate levels inhibit the activation of the NOD‐like receptor family pyrin domain‐containing protein 3 (NLRP3) inflammasome and cysteine‐aspartic protease 1 (Caspase‐1), thereby suppressing pyroptosis [38]. Conversely, in acute liver injury (ALI), lactate enhances the activation of Gasdermin D (GSDMD) protein, accelerates macrophage pyroptosis and exacerbates liver damage [39]. Furthermore, in sepsis models, elevated lactate levels can induce lymphocyte and human kidney 2 (HK‐2) cell apoptosis by activating the programmed cell death protein 1 (PD‐1)/programmed cell death ligand 1 (PD‐L1) pathway, leading to immunosuppression [40, 41]. In tumour cells, regulating LDH can interfere with cell survival through multiple pathways, including apoptosis, pyroptosis and autophagy [42]. Therefore, the effects of lactate on cellular injury are complex and cannot be simply categorised as either promoting or inhibiting. The impact of lactate on various cell death pathways is complex and requires further research for a deeper understanding.

### 3.5 Other Biological Processes

Lactate regulates memory formation, neuroprotection and wound healing. Notably, lactate is one of the most crucial factors in wound healing, and its salts are commonly used in clinical practice to treat nonhealing wounds and promote tissue repair [43]. It can also stimulate neuronal plasticity, enhance memory and provide neuroprotection [44]. Although these functions are noteworthy, they are beyond the scope of this article, so further discussion is not provided.

## 4 Lactate Metabolism in the Kidneys

Intracellular lactate is derived from both intracellular production and extracellular uptake. The intracellular lactate generation depends on the balance between glycolysis and mitochondrial metabolism. The transport of lactate across the cell membrane is primarily mediated by monocarboxylate transporters (MCTs), with lactate being the primary substrate for MCT1–4 [45]. Lactate signalling is conducted mainly through GPR81 [46, 47]. This receptor is widely expressed across various tissues and organs, where it mediates the role of lactate in energy regulation, immunosuppression and tumour growth [10, 11]. Intracellular lactate has two primary fates: it can be oxidised to pyruvate and enter the TCA cycle to produce energy, or it can be converted into energy substrates through gluconeogenesis in the liver and kidneys. The discovery of lactylation revealed a novel pathway for lactate utilisation in cellular processes. The kidneys, which participate in both glycolysis and gluconeogenesis, play a central role in lactate metabolism, consuming lactate while simultaneously producing it and regulating glucose metabolism [48, 49]. Consequently, the kidneys are regarded as a primary systemic reservoir for lactate.

**FIGURE 2:**
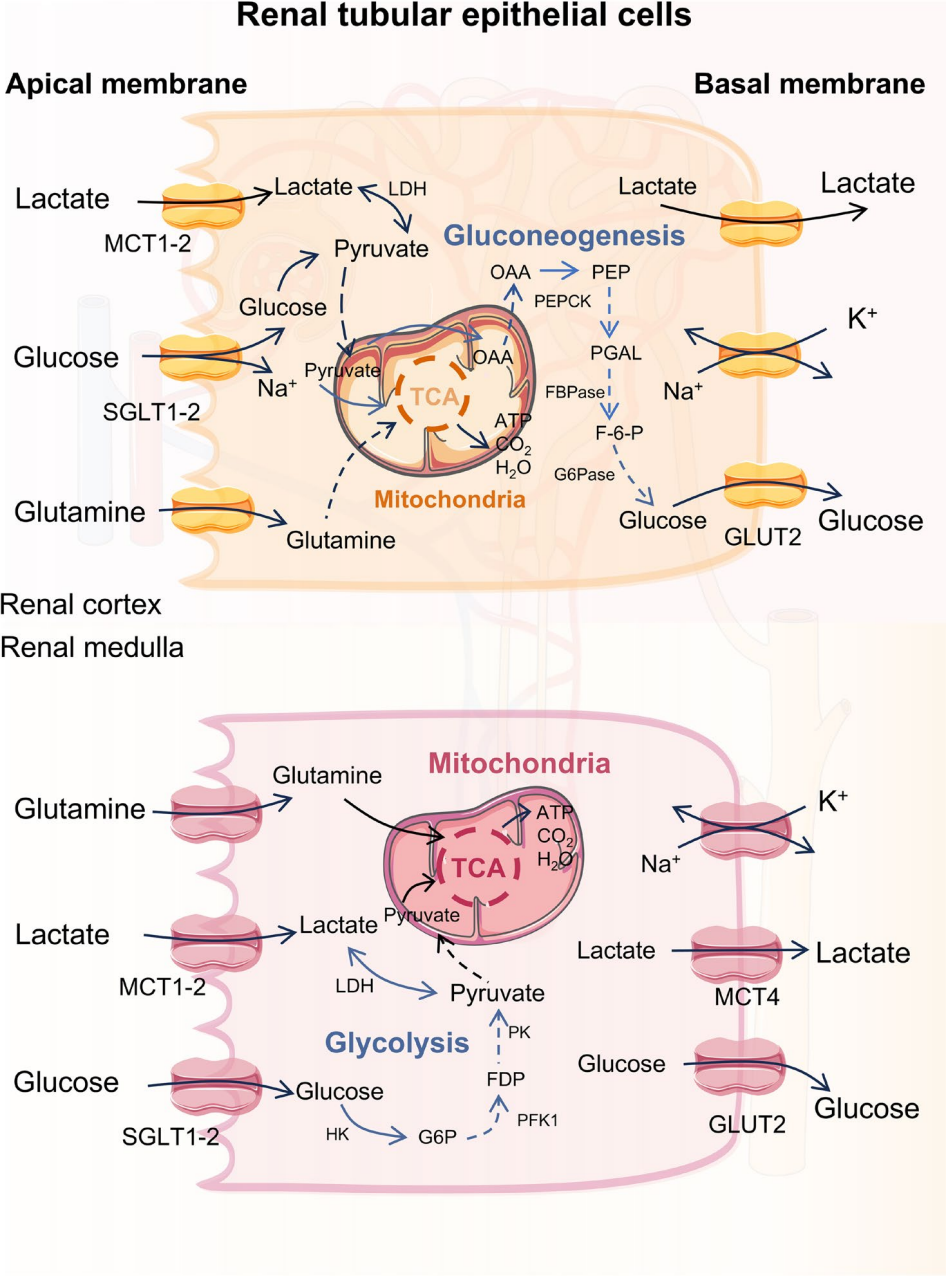
Cortex–medulla glucose–lactate cycle in the kidneys. Lactate reabsorption primarily occurs in the renal cortex, especially in tubular epithelial cells. Lactate is oxidised to produce energy and serves as a substrate for gluconeogenesis. Most of the glucose that is produced in the cortex will be transported to the medullary tubular epithelial cells and further utilised to produce lactate.

Current studies on renal lactate metabolism are primarily performed by measuring arteriovenous differences in lactate concentration and isotope tracing techniques. Lactate in the bloodstream is completely filtered by the glomeruli and almost entirely reabsorbed by proximal tubular epithelial cells (PTECs). Consequently, the amount of lactate excreted in urine is minimal, except for extremely elevated lactate levels [50]. The kidneys exhibit a unique cortex–medulla glucose–lactate cycle (Figure 2). Lactate reabsorption mainly occurs in the renal cortex, where it is oxidised to generate energy and produces glucose, which is then released back into the medulla. The renal medulla primarily consumes glucose and produces lactate. This cycle aligns with the distinct material transport requirements between the cortex and medulla. Notably, lactate production is reduced by 39% and 50%, respectively, when Na^+^ reabsorption is inhibited or when glomerular filtration is completely blocked. This phenomenon indicates that glycolysis is essential for Na^+^ transport in the medulla [51, 52]. In addition, renal medullary glucose metabolism relies primarily on glycolysis, even under adequate substrate and oxygen supply [53]. This feature is consistent with the ability of the medulla to concentrate urine, which requires substantial energy to counteract concentration gradients. Lactate production is directly related to renal function, and glucose utilisation is necessary to maintain renal function.

Renal gluconeogenesis is influenced by various factors. Lactate can constitute up to 50% of the energy sources for renal gluconeogenesis, which becomes particularly important during acute illnesses. During fasting, hypoglycaemia, elevated circulating adrenaline and exercise, the renal contribution to circulating glucose through gluconeogenesis can significantly increase from 5%–16% to 40% [50, 51, 54]. In cases of exogenous hyperlactataemia, renal lactate clearance reaches 20%–30% [48, 49]. Owing to the restricted distribution of key enzymes, renal gluconeogenesis primarily occurs in PTECs [50, 55]. This process provides energy to tubular cells while reducing lactate concentration, thereby maintaining acid**–**base balance.

Various pathological conditions, including massive haemorrhage and acid**–**base imbalances, can affect the ability of the kidneys to process lactate. However, the kidneys exhibit remarkable resilience, as evidenced by canine studies where lactate clearance ceases only upon a reduction of 90% or more in renal blood flow. Severe acidosis significantly inhibits hepatic lactate uptake, whereas it enhances renal lactate metabolism, partially compensating for the loss of hepatic lactate metabolism [56, 57]. Despite the crucial role of the kidneys in lactate metabolism, the mechanisms underlying different pathophysiological conditions in the kidneys remain unclear. The fate of lactate within the kidneys is complex and depends on various hormones and physiological stimuli, with differences between the cortex and medulla. AKI or CKD can impair renal lactate processing, leading to abnormal lactate accumulation in the body. Recent studies indicate that the genetically encoded fluorescent lactate sensor, FiLa, offers detailed insights into lactate metabolism at multiple levels, positioning it as a promising tool for metabolic research in kidney diseases [58]. Further investigation is required to fully assess its effectiveness.

## 5 The Role of Lactate in AKI‐to‐CKD Progression

AKI involves complex interactions among inflammation, microcirculatory dysfunction and metabolic reprogramming, making its pathogenesis intricate [12]. During the early stages of AKI, PTECs undergo metabolic reprogramming, which is characterised by increased glycolysis. The loss of gluconeogenesis represents a key metabolic phenotype in AKI, as evidenced by elevated lactate levels in AKI models induced by sepsis, ischemia–reperfusion (I/R) injury, glycerol, LPS and folic acid (FA; Table 1). In these AKI models, lactate is also identified as a risk factor, playing a role in the onset and progression of the disease [14, 40, 41, 59, 60]. Lactate levels are positively correlated with mortality in patients with AKI, with significantly higher serum lactate concentrations in nonsurvivors than in survivors [61]. Elevated lactate is an independent risk factor for sepsis‐associated AKI (SAKI), where patients with higher lactate levels (≥ 4 mmol/L) experience significantly increased AKI incidence and a greater need for renal replacement therapy [62, 63]. Conversely, a higher lactate clearance rate within the first 24 h of admission is associated with better renal outcomes in critically ill patients.

**TABLE 1:** Studies on lactate in various types of AKI.

Diseases	Influence	Animal models	Cell types	Patients
SAKI	Downregulated SIRT3 and p‐AMPK expression inhibits autophagy, increases apoptosis and exacerbates SAKI	CLP‐induced septic mouse model	HK‐2 cells	—
SAKI	Activated PD‐1/PD‐L1 signalling induces lymphocyte apoptosis, leading to immunosuppression and worsening SAKI	CLP‐induced septic mouse model	TCMK‐1	—
SAKI	Induced mitochondrial dysfunction exacerbates SAKI	CLP‐induced septic mouse model	HK‐2 cells	—
SAKI	Elevated expression of HCA2 promotes M2 macrophage polarisation to alleviate inflammation. In addition, it partially improves mitochondrial function	CLP‐induced septic mouse model	—	—
SAKI	Leads to mitochondrial dysfunction and cellular senescence	Albicans‐induced SAKI mouse model	HK‐2 cells	—
LPS‐AKI	Increases cellular inflammation and apoptosis, worsening AKI	LPS‐induced AKI mouse model	HK‐2 cells	—
LPS‐AKI	Exosomes from adipose‐derived stem cells inhibit aerobic glycolysis, suppressing apoptosis and inflammation and alleviating AKI	LPS‐induced AKI mouse model	HK‐2 cells	—
I/R‐AKI	The USP25‐PKM2‐aerobic glycolysis axis promotes M1‐like macrophage polarisation and proinflammation, exacerbating AKI	I/R‐induced AKI mouse model	BMDMs	ATIN patients
FA‐AKI	Elevated NRK‐49F cell activation and HGF expression facilitate the proliferation and regeneration of ECs, promote renal function recovery	FA‐induced AKI mouse model	NRK‐49F	—
AKI‐to‐CKD	Drives renal interstitial fibrosis progression from AKI to CKD	IRI, UUO‐induced AKI‐to‐CKD mouse models	Kidney pericyte	—

*Note*: — denotes studies in which research has not been conducted at the cellular or clinical level.

In AKI, lactate can inhibit autophagy, enhance apoptosis and cellular senescence, induce immunosuppression, disrupt mitochondrial function and promote cellular inflammation, ultimately compromising kidney function. Reducing lactate levels is shown to provide a degree of renoprotection. However, several studies have demonstrated that lactate can exert some renoprotective effects by promoting macrophage phenotype transformation, suppressing inflammatory responses and facilitating the proliferation and regeneration of epithelial cells [14, 40, 63, 64, 65, 66, 67, 68, 69]. Lactate has been reported to exert contradictory regulatory effects on AKI. These opposing actions are likely influenced by multiple factors, such as variations in cell models, disease stages, lactate concentrations and the specific pathways involved. These findings underscore the complexity of the role of lactate in AKI. Therefore, further studies are required to elucidate the underlying mechanisms involved.

Lactate plays a crucial role in the transition from AKI to CKD. After AKI, lactate promotes the fibrotic process by regulating the metabolic state of endothelial and stromal cells. Its accumulation not only exacerbates renal injury in AKI but also accelerates the AKI‐to‐CKD progression. In the I/R‐induced AKI‐CKD model, lactate is found to promote the transdifferentiation of pericytes into myofibroblasts, thereby contributing to renal interstitial fibrosis during the transition from AKI to CKD [70].

Early‐stage CKD often involves impaired glucose metabolism, reduced gluconeogenesis and increased lactate, which are correlated with disease severity (Table 2) [71]. Irreversible fibrosis is a common feature of CKD pathology. During renal fibrosis, metabolic reprogramming in RTECs leads to hyperlactatemia [72, 73]. The lactate/albumin (L/A) ratio shows a nonlinear, progressively increasing association with mortality in CKD patients, helping to identify individuals at high risk for all‐cause mortality [74]. In DN, the degree of lactate elevation is closely related to urinary albumin levels. Notably, angiotensin II receptor blocker therapy can reverse elevated lactate levels in patients with DN, consequently reducing proteinuria and alleviating renal pathology [75, 76, 77]. Various CKD models, including the unilateral ureteral obstruction (UUO), I/R, FA‐induced renal fibrosis, ablation and infarction (A/I), adenine‐induced CKD, STZ‐induced and db/db DKD mouse models, as well as models of polycystic kidney disease (PKD) and hypertensive nephropathy, have consistently demonstrated that lactate accumulation activates the transcription factor hypoxia‐inducible factor 1‐alpha (HIF‐1α) and pathways such as mechanistic target of rapamycin complex 1 (mTORC1). These processes regulate oxidative stress and inflammation, thereby exacerbating renal fibrosis and impairing kidney function [39, 78, 79, 80, 81, 82, 83, 84, 85, 86].

**TABLE 2:** Studies on lactate in various types of CKD.

Diseases	Influence	Animal models	Cell types
ADPKD	Promotes M2 macrophage polarisation and excessive polyamine production, worsening renal fibrosis in ADPKD	Dehydration‐induced PKD mouse model	CLECs, RTECs
CKD	Worsens renal damage by altering pH and activating NF‐κB signalling	—	UMR106
CKD	Upregulated PROM1 in RTECs suppresses glycolysis and mitigates renal fibrosis	UUO‐induced renal fibrosis mouse model	HK‐2 cells
CKD	3‐BP inhibits lactate synthesis by suppressing the IRAK4‐MYC axis, providing renoprotection	UUO‐induced renal fibrosis mouse model	NRK‐49F
CKD	PHD inhibitors reduce lactate and improve survival rates in the CKD model	MALA‐induced CKD mouse model	—
DN	Increase in renal inflammation exacerbates renal damage	STZ‐induced and db/db DKD mouse models	HUVECs, THP‐1 cells
DN	Suppression of lactate elevation ameliorates renal fibrosis and provides renoprotection	STZ‐induced and db/db mouse models	HK‐2 cells
HN	Elevated lactate levels activate the TRPV4‐TGFβ1‐SMAD2/3‐CTGF‐mediated renal fibrosis pathway, exacerbating HN	Spontaneously hypertensive rats	NRK‐49F
RIF	Induced proliferation of NRK‐49F cells exacerbates renal interstitial fibrosis	5/6 A/I‐induced CKD mouse model	NRK‐52E, NRK‐49F
RIF	Activation of mTORC1 exacerbates renal fibrosis by increasing lactate	UUO, I/R, FA‐induced fibrosis mouse model	NRK‐52E

*Note*: — denotes studies in which research has not been conducted at the cellular or animal level.

Lactate measurement in blood and urine has now become a routine clinical practice, providing valuable diagnostic insights into various conditions, including inflammatory diseases, neoplastic disorders and ischemic–hypoxic injuries. Currently, most studies on lactate in kidney diseases focus on its role in clinical diagnosis and treatment. The current mechanistic studies on AKI‐to‐CKD progression are limited and are primarily based on animal and cell models with minimal clinical validation. Compared with AKI, studies on lactate in CKD have yielded consistent findings. These findings suggest that the mechanism of action of lactate may vary across different stages and types of kidney disease. In the early stages of AKI, lactate has complex effects, potentially providing protection by promoting cell regeneration and modulating immune inflammation. As kidney injury progresses, lactate accumulation may induce adverse effects such as immune dysregulation, oxidative stress and mitochondrial dysfunction, thereby exacerbating kidney damage, promoting fibrosis and eventually leading to CKD. In the AKI‐to‐CKD and CKD, kidney function is already severely impaired, and lactate accumulation likely leads to more severe metabolic abnormalities, inflammatory responses and kidney dysfunction, further accelerating disease progression. The precise role of lactate in the AKI‐to‐CKD progression remains unclear and warrants further investigation.

## 6 The Role of Lactylation in the AKI‐to‐CKD Progression

With the rapid advancement of HPLC‐MS, various novel acylation pathways have been discovered. In 2019, Zhang et al. first identified the existence of histone lysine lactylation after analysing core histones in human MCF7 cells. They also demonstrated that histone lactylation exhibits unique temporal dynamics, suggesting that this modification might act as a ‘lactate clock’ during macrophage phenotype switching, promoting the macrophage transition from M1 to M2 [2]. Subsequent studies have revealed that lactylation also occurs in nonhistone proteins, where it performs a regulatory role [87]. Studies have indicated that lactylation is modulated by various substances through lactate level regulation, suggesting that lactate molecules solely influence lactylation. LDH knockout completely abolishes lactylation, distinguishing it from other acylation pathways. Table 3 summarises the substances and related mechanisms that can regulate lactylation upstream of lactylation pathways.

**TABLE 3:** Substances affecting lactylation and their targets.

Regulatory mechanism	Regulatory substances or targets	Impact
Enhanced glycolysis	Aldolase B, BZW2, BEV, BRAFV600E‐mutant, KLK2, PDK1/2, BY4741, STAT5, VEGF, Zeb1	↑
Increased LDHA activity	AKR1B10, KLF15, PM2.5, ULK1	↑
Unknown mechanisms	Huazhuo Tiaozhi granule, Dux, LPS	↑
Activation of lactylation enzymes	ZEB1, High levels of copper	↑
Activation of the PI3K/AKT pathway	TNF‐α, PCSK9	↑
Reduced LDHB expression	APAP	↑
Activation of the Hippo pathway	GPR37	↑
Increased lactate influx	MCT1	↑
Increased HIF‐1α expression	HSPA12A	↑
Glycolysis inhibition	2‐DG, COP, MCT1 inhibitor 7ACC, OXA, RJA, SalB, STP, AST‐120	↓
Unknown mechanisms	DMZ, HSP12A, HPV16 E6, MPC1, MUC20	↓
Increased delactylation	20 (S)‐Rh2, GA, HNL	↓
Lactylation enzyme inhibition	Andrographolide, β‐alanine	↓
Increased OXP	DCA, IDH3β	↓
Activation of the Hippo pathway	CircXRN2	↓
Activation of Wnt/MEK pathway	TRA	↓
Inhibition of HIF‐1α	Dex	↓
Increased LDHB synthesis	PGC‐1α	↓
Increased lactate efflux	MCT4	↓
Enhanced p53 activity	BMP	↓
Blockade of the PI3K/AKT pathway	RGS5	↓

*Note*: ↑ denotes substances or targets that promote lactylation. ↓ denotes substances or targets that inhibit lactylation.

In the lactylation process, the specific enzymes or complexes that control the addition or removal of lactylation are termed ‘writers’ and ‘erasers’, respectively, whereas the effector proteins that read subsequent transcriptional signals are known as ‘readers’. Once these signals have been read, they can influence downstream signalling pathway activation, initiating various biological events [88]. The enzymes that act as ‘writers’ and ‘erasers’ are histone acetyltransferase (HAT) p300 and deacetylases, including histone deacetylases (HDACs) and Sirtuin (SIRT) 1–3, in the process of histone lactylation [2, 78]. HDACs are the primary enzymes involved in the delactylation process [79, 80]. Enzymes identified as lactylation regulators also regulate other PTMs, including acetylation, butyrylation and succinylation. Specific regulatory enzymes involved in histone lactylation remain elusive, indicating that different acetylation pathways may interact within cells or tissues (Table 4).

**TABLE 4:** Studies on histone lactylation.

Targets	Diseases	Mechanisms	Remarks
H3K18la	ASCVD	Activates the transcription of anti‐inflammatory and TCA cycle genes, initiating local repair and homeostasis	※
	BLCA	Drives the expression of YBX1 and YY1, promoting cisplatin resistance in BLCA	
		CircXRN2 triggers Hippo signalling, suppressing H3K18la‐driven tumour progression, and is downregulated in BLCA	
	CRC	Upregulates RUBCNL expression, enhances autophagy and leads to cancer cell survival	
		Suppression of RARγ transcription in macrophages activates the TRAF6‐IL‐6‐STAT3 pathway	
		GPR37 increases H3K18la through Hippo signalling activation, upregulates CXCL1 and CXCL	
		NSUN2‐ENO1‐H3K18la forms a positive feedback loop that drives CRC progression	
	DN	Leads to FTO upregulation, initiating crosstalk among vascular endothelial cells, pericytes, and microglia	
	EC	Enhances USP39 expression, modulating the PI3K/AKT/HIF‐1α pathway to promote endometrial cancer progression	
	EMs	HMGB1 upregulation activates MAPKs and NF‐κB signalling, driving EM progression	
	GC	VCAM1 transcription in GC cells is activated, enhancing AKT–mTOR‐CXCL1 pathway, recruiting hGC‐MSCs	
		GLUT3 overexpression enhances lactylation, driving tumour progression	
	GBM	Upregulation of NF‐κB signalling enhances histone lactylation, activates LINC01127 expression	
		Expression of CD39, CD73, and CCR8 is increased, boosting immunosuppression and diminishing CAR‐T efficacy	
	Inflammation	Promotes the conversion of Th17 cells to Tregs, exerting an immunosuppressive effect	
		Metformin reduces H3K18la levels, inhibits ROS production, and mitigates inflammatory damage	
	I/R injury	Activates YTHDF1/m6A/NREP, promoting the conversion of fibroblasts to myofibroblasts	
	Liver diseases	ALDOA expression is regulated by IGF2BP2, which increases lactylation, activates HSCs, and induces liver fibrosis	
		Affects M1 polarisation of macrophages	
		Deletion of HK2 expression in HSCs inhibited H3K18la, alleviated HSCs activation and liver fibrosis	
	LC	Reduced expression of SLC25A29 increases EC proliferation and migration.	
		BZW2‐mediated upregulation of H3K18la at the IDH3G promoter advances LUAD progression	
		Promotes neuroendocrine differentiation in lung cancer and resistance to targeted therapies	
	PF	Activates YTHDF1/m6A/NREP, promoting the transformation of fibroblasts into myofibroblasts.	
	PH	The mROS‐HIF‐1α axis elevates H3K18la levels, promotes PASMC proliferation and leads to PH	
H3K18la
	MPE	FOXP3 NKT‐like cells highly express MCT1 and LDH, maintaining H3K18la levels and immunosuppressive functions	
	MI	Promotes the transcription of repair genes and the anti‐inflammatory and pro‐angiogenic activities of macrophages	※
	Myopia	Activates Notch1 transcription, promoting the conversion of fibroblasts to myofibroblasts	
	OM	Enhances ALKBH3 expression, weakens PML body formation and promotes malignant transformation of cancer	
	OP	Promotes BMSCs differentiation into osteoblasts, improving OP	※
	PCa	Promotes neuroendocrine differentiation in lung cancer and resistance to targeted therapies	
		Zeb1 promotes histone lactylation, induces neural gene expression and advances NEPC progression	
		Inhibiting the phagocytic activity of macrophages leads to drug resistance	
		PI3Ki inhibits H3K18la, enhances the phagocytic ability of activated TAMs, overcoming immune evasion	
	SAKI	Leads downstream inflammation and cell apoptosis	
	SS	Mediating the overexpression of inflammatory cytokines and Arg1 stimulates the functions of macrophage	
	BC	c‐Myc‐SRSF10 axis upregulation drives selective splicing of MDM4 and Bcl‐x in cancer cells, resulting in BC	
	ccRCC	H3K18la and PDGFRβ create a positive feedback loop, driving disease development	
	UC	*S. cerevisiae* BY4741 modulates gut microbiota via H3K18la, treating UC	※
	AD	Forms IDH3β‐PAX6‐IDH3β positive feedback loop	
PDAC	Forms glycolysis‐H3K18la‐TTK/BUB1B positive feedback loop	
H3K9la	ESCC	Promotes LAMC2 expression, thereby enhancing ESCC invasiveness	
	GBM	The upregulation of LUC7L2, reduction of MLH1, and inhibition of mismatch repair result in TMZ resistance in GBM	
	HCC	RJA suppresses H3K9la, inhibiting HCC progression	
	GC	GLUT3 drives EMT by modulating lactylation in GC, resulting in metastasis and increased invasiveness	
H4K12la	AD	Forms glycolysis‐H4K12la‐PKM2 positive feedback	
	ATC	Activation of multiple genes essential for ATC proliferation	
	CKD	Activation of the NF‐κB signalling pathway leads to kidney inflammation and fibrosis	
	LC	Forms AKR1B10‐glycolysis‐H4K12la‐CCNB1	
H3K14la	DN	Upregulated KLF5 inhibits E‐cadherin expression and thus promotes EMT in DN.	
	LUAD	Decreased SLC25A29 expression increases EC proliferation and migration	
H4K5la	AML	Induced transcription of PD‐L1 drives immune suppression in AML	
	GC	GLUT3 drives EMT by modulating lactylation in GC, resulting in metastasis and increased invasiveness	
H4K8la	CRC	Enterobacter LPS increases lactylation levels, reduces YY1 binding efficiency, upregulates LINC00152 expression	

*Note*: ※ denotes studies on the protective effects of lactylation against progressive diseases.

Nonhistone lactylation, which was discovered recently, remains poorly understood, particularly regarding its molecular mechanisms. Researchers hypothesise that it occurs primarily through nonenzymatic pathways, with lactylglutathione acting as an essential intermediate [79, 81]. Recent studies have revealed that the aminoacyl‐tRNA synthetase 1/2 (AARS1/2) and acetyltransferase Tat‐interacting protein 60 (TIP60) are closely linked to nonhistone lactylation [82, 83]. Additionally, in *Escherichia coli* , YiaC has been identified as an enzyme capable of catalysing lactylation, whereas cobalamin‐dependent protein (CobB) can remove this PTM both in vitro and in vivo [84]. However, the involvement of these enzymes in other types of protein modifications remains unclear and warrants further investigation (Table 5).

**TABLE 5:** Studies on nonhistone lactylation.

Targets	Diseases	Mechanisms	Remarks
ACSF2 K182la	DN	Induces mitochondrial dysfunction, accelerating DN advancement	
ALDOA K147la	CRC	Unclear	
PFKP K688la	CRC	Unclear	
AMPKα la	IDD	Increased lactylation of AMPKα enhances NP cell senescence and exacerbates IDD	
CACNA2D1 la	GDM	Involvement in histone lactylation‐mediated PI3K‐Akt, JAK–STAT, and mTOR pathways exacerbates the disease	
CCNE2 K348la	HCC	Reduced apoptosis in HCCs promotes HCC progression	
CENPA K124la	HCC	Activation of CENPA drives the expression of CCND1 and NRP2, promoting HCC progression	
CNPY3 la	PCa	GBA delactylates CNPY3 through IRT1, promoting lysosomal rupture, triggering cell pyroptosis	※
DCBLD1 K172la	CxCa	Activation of the pentose phosphate pathway promotes cervical cancer progression	
eEF1A2 K408la	CRC	Increased translation elongation rates enhance protein synthesis and promote tumorigenesis	
Fis1 K20la	AKI	Interacts with DRP1 to induce excessive mitochondrial fission, worsening AKI	
G6PD K45la	CxCa	HPV16 E6 inhibits G6PD K45la and activates the pentose phosphate pathway, leading to cervical cancer progression	※
Hepatocyte K67la	NAFLD	MPC1 knockout increases fatty acid synthase K673 la, inhibits its activity, and reduces hepatic lipid deposition	
HIF1α la	PCa	Increased transcription of KIAA1199 promotes angiogenesis and tumour progression	
HMGB1 la	LI/R injury	Increased macrophage chemotaxis and inflammatory activation lead to liver I‐R injury	
	Sepsis	It is released into the circulation via exosomes, disrupting endothelial integrity and increasing vascular permeability	
IGF‐1R la	MM	MUC20 inhibits CDKN2A la, IGF‐1R la and induces cuproptosis, weakening PI resistance in MM cells	
Mecp2 k271la	ASCVD	Reduced Ereg expression and MAPK activity promotes atherosclerosis regression	
METTL16 K229la	GC	Lactate transferase AARS1/2 promotes METTL16 K229la, induces cuprptosis, and leads to disease progression	
METTL3 la	APL	GRh2 inhibits METTL3 la, improving ATRA resistance in APL	
MOESIN 72la	Cancer	Enhancing Treg function promotes tumour progression	
MRE11 K673la	Cancer	Promoting DNA binding, end resection, and HR increases chemotherapy resistance	
NAT10 K290la	KSHV infection	Leads to tRNA^Ser‐CGA‐1‐1^ ac^4^C, promoting KSHV reactivation	
NEDD4 K33la	AILI	Enhancing GSDMD activation accelerates pyroptosis, leading to liver injury	
NMNAT1 K128la	PAAD	Promotes nuclear translocation, maintains NMNAT1 activity, and promotes cancer cell survival	
NSUN2 K356la	CRC	NSUN2‐ENO1‐H3K18la positive feedback loop promotes CRC progression	
NUSAP1 K34la	PDAC	NUSAP1‐LDHA‐Glycolysis‐Lactate positive feedback promotes malignant phenotype in PDAC	
p53 K120la, p53 K139la	Cancer	AARS1 weakens the tumour‐suppressive function of p53 via p53 K120 la and p53 K139la	
RIG‐I K852la	CRLM	Inhibiting NF‐κB signalling affects Tregs and CD8^+^ T cell activity, promoting tumour progression	
SHMT2 la	EC	Enhanced MTHFD1L expression accelerates EC progression	
Snail1 la	MI	Activating the TGF‐β/Smad2 pathway increases cardiac fibrosis	
Sox10 la	Inflammation	TNF‐α induces Sox10 la via PI3K/AKT signalling, driving VSMC transdifferentiation and pyroptosis	
VEGFR2 la, VE‐cadherin la	GBM	Unclear	
YY1 K183la	AU	Upregulates the secretion of inflammatory factors, promoting microglia migration and proliferation	
	ROP	Enhancing FGF2 transcription and promoting accelerated angiogenesis leads to blindness	
α‐MHC K1897la	HF	α‐MHC K1897la alters the interaction between α‐MHC and Titin, mitigating heart failure	※

*Note*: ※ denotes studies on the protective effects of lactylation against progressive diseases.

**FIGURE 3:**
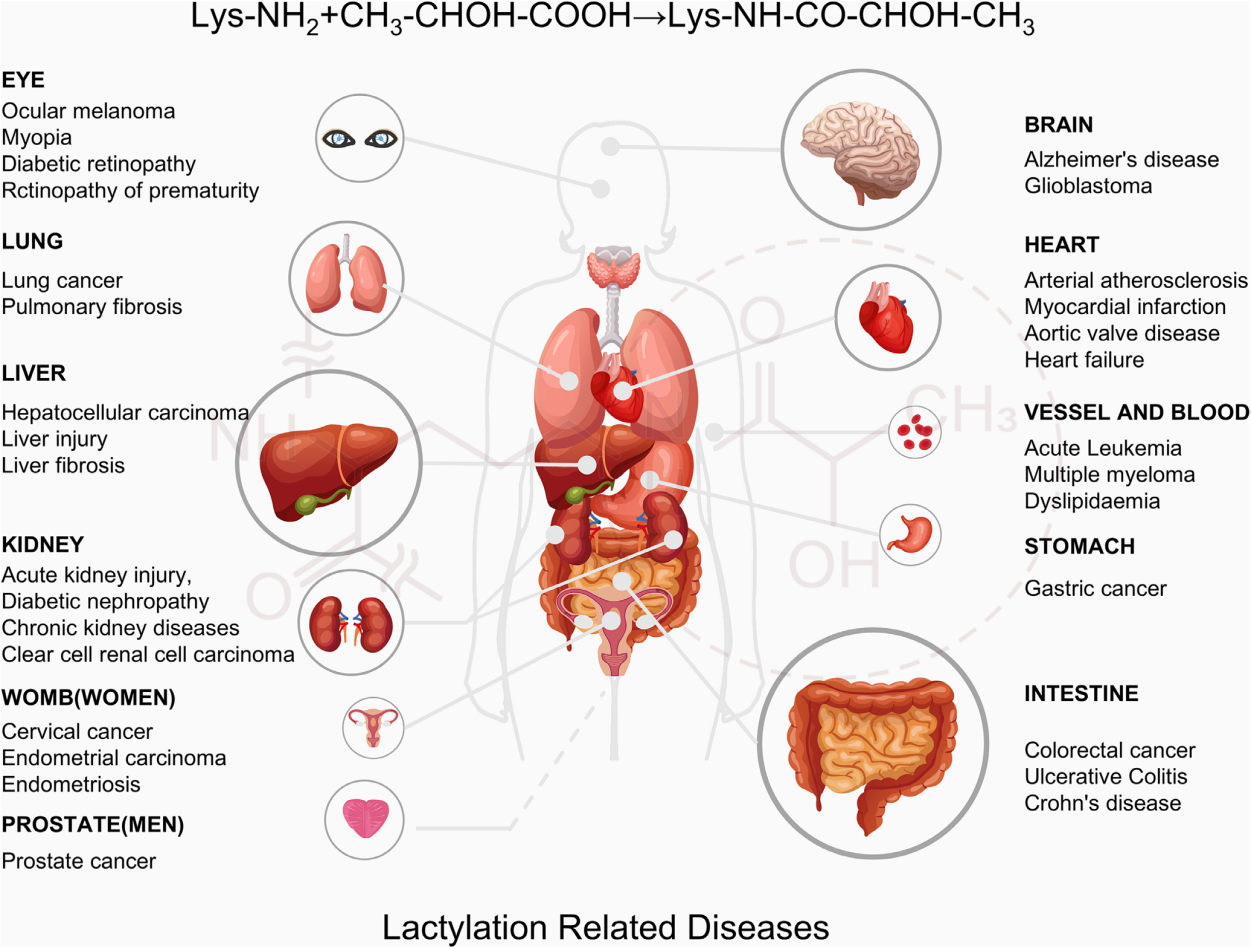
Lactylation is involved in various human diseases. This figure summarises studies on different types of organ damage and diseases closely associated with lactylation. In this figure, the size of each organ visually represents the frequency of research, arranged in descending order: Gastrointestinal tract, liver, brain, lungs, heart, kidneys, eyes, prostate, blood and uterus. Each organ is annotated with the related diseases that have been studied. This section of overview encompasses both histone and nonhistone lactylation, which provides a reference for further exploration of lactylation under various conditions.

A literature review on lactylation revealed that it has extensive regulatory effects on both health and disease, affecting multiple organs, including the gastrointestinal tract, liver, brain, lungs, heart, kidneys, eyes, prostate, blood and womb (Figure 3). It is involved in multiple pathological processes, such as tumour progression, I/R injury, immune‐related inflammation, organ fibrosis and neuroembryonic development. Lactylation also regulates various cell death pathways, including pyroptosis, ageing, autophagy and cuproptosis [38, 39, 85, 86, 89, 90, 91, 92, 93, 94]. Currently, research on lactylation is most extensive in the field of oncology [95, 96]. It has been implicated in the development of several cancers, such as colorectal cancer (CRC), hepatocellular carcinoma (HCC), prostate cancer (PCa), glioblastoma, leukaemia and clear cell renal carcinoma (ccRCC) [94, 97, 98, 99, 100, 101, 102, 103, 104, 105, 106, 107, 108, 109, 110, 111]. Current evidence suggests that increased lactylation in the body generally accelerates disease progression, although some studies have demonstrated that it can have protective effects. For instance, lactylation improves coronary atherosclerosis and heart failure prognosis and prevents osteoporosis and dyslipidaemia. Moreover, lactylation induced by physical exercise may help to prevent certain diseases [91, 112, 113, 114].

Several studies have revealed the presence of positive feedback regulatory loops within the body that contribute to rapid disease progression. Notably, specific loops have been identified in various diseases: The NOP2/Sun RNA methyltransferase 2 (NSUN2), enolase 1 (ENO1) and histone H3 lysine 18 lactylation (H3K18la) loop in CRC; the nucleolar spindle‐associated protein 1 (NUSAP1)‐LDHA‐lactylation and H3K18la‐threonine tyrosine kinase (TTK)/BUB1 mitotic checkpoint serine/threonine kinase B (BUB1B) loops in PC; the platelet‐derived growth factor receptor β (PDGFRβ)‐H3K18la loop in ccRCC; and the isocitrate dehydrogenase 3 beta (IDH3β)‐lactylation‐Paired box 6 (PAX6) and histone H4 lysine 12 lactylation (H4K12la)‐pyruvate kinase M2 (PKM2) loops in Alzheimer's disease [68, 97, 111, 115, 116, 117, 118]. The most extensively studied target is H3K18la [89, 94, 119, 120, 121, 122, 123], due to its dual role as a marker for active promoters and tissue‐specific active enhancers [124]. Its prominence may also be influenced by technical factors, such as antibodies targeting this site, which have been more advanced and widely utilised (Tables 4 and 5).

This discovery introduced a new field of study in protein PTMs, revealing potential mechanisms by which lactate regulates cellular metabolism and function and linking metabolism with gene expression. These findings suggest new directions for understanding the mechanisms underlying various metabolic disorders, including immune‐related inflammation, tumour proliferation and hypoxia injury [8, 33, 125, 126, 127]. Lactylation also provides novel insights into AKI‐to‐CKD mechanisms. However, several questions remain unanswered. For instance, the validity of enzymatic versus nonenzymatic classifications of lactylation in vivo remains unclear. In the lactylation process, the differences between l‐lactate and d‐lactate are poorly understood, and it remains unknown whether the resulting protein modifications have consistent functions. Additionally, how nonspecific enzymes can specifically catalyse different acyl modifications on histones remains a significant question. Many of the proposed mechanisms are still speculative.

The kidney, as a key organ for lactate regulation, has an unclear regulatory mechanism regarding lactylation. Studies on lactylation in kidney diseases are scarce and have primarily focused on AKI‐to‐CKD (Table 6). Based on established regulatory roles and related signalling pathways of lactylation, combined with previous studies on lactate in AKI‐to‐CKD, potential mechanisms underlying these processes can be hypothesised and subsequently validated through further studies.

**TABLE 6:** Mechanisms associated with lactylation in kidney diseases.

Diseases	Targets	Mechanisms	Animal models	Cell types	Patients
AKI	Fis1 K20la	Fis1 interacts with DRP1 to promote excessive mitochondrial fission	CLP‐induced SAKI mouse model	LPS‐stimulated HK‐2 cells	Sepsis patients
AKI	H3K18la	Activate RhoA/ROCK1/Ezrin signalling and Ezrin K263 lactylation, leading to inflammation and apoptosis	CLP‐induced SAKI mouse model	LPS‐stimulated HK‐2 cells, PCTECs	—
AKI	H3K18la	HK2‐H3K18la forms a positive feedback loop, exacerbating renal IRI	I/R‐induced AKI mouse model	H/R‐induced HK‐2 cells	—
ccRCC	H3K18la	H3K18la and PDGFRβ form a positive feedback loop, promoting disease development	PDX mouse	HK2 cells, human RCC cell lines	—
ccRCC	Histone	Interfere with m6A DEGs to reveal prognostic and TME characteristics of ccRCC	—	HK‐2 and ACHN cells	—
CKD	H4K12la	Enhanced transcription of NF‐κB signalling related genes in PTC leads to kidney inflammation and fibrosis	IRI, FA, H/R‐induced CKD mouse model	—	CKD patients
DN	ACSF2 K182la	Cause mitochondrial dysfunction	db/db mouse, db/m mouse	HK2 cells	DN patients
DN	H3K14la	Elevated expression of KLF5 recognises the cdh1 promoter, suppresses the expression of E‐cadherin and accelerates EMT in DN	db/db mouse, db/m mouse	HK2 cells, mTECs	—

*Note*: — denotes studies in which research has not been conducted at the cellular, animal, or clinical level.

### 6.1 Effects of Histone Lactylation in the AKI‐to‐CKD Progression

**FIGURE 4:**
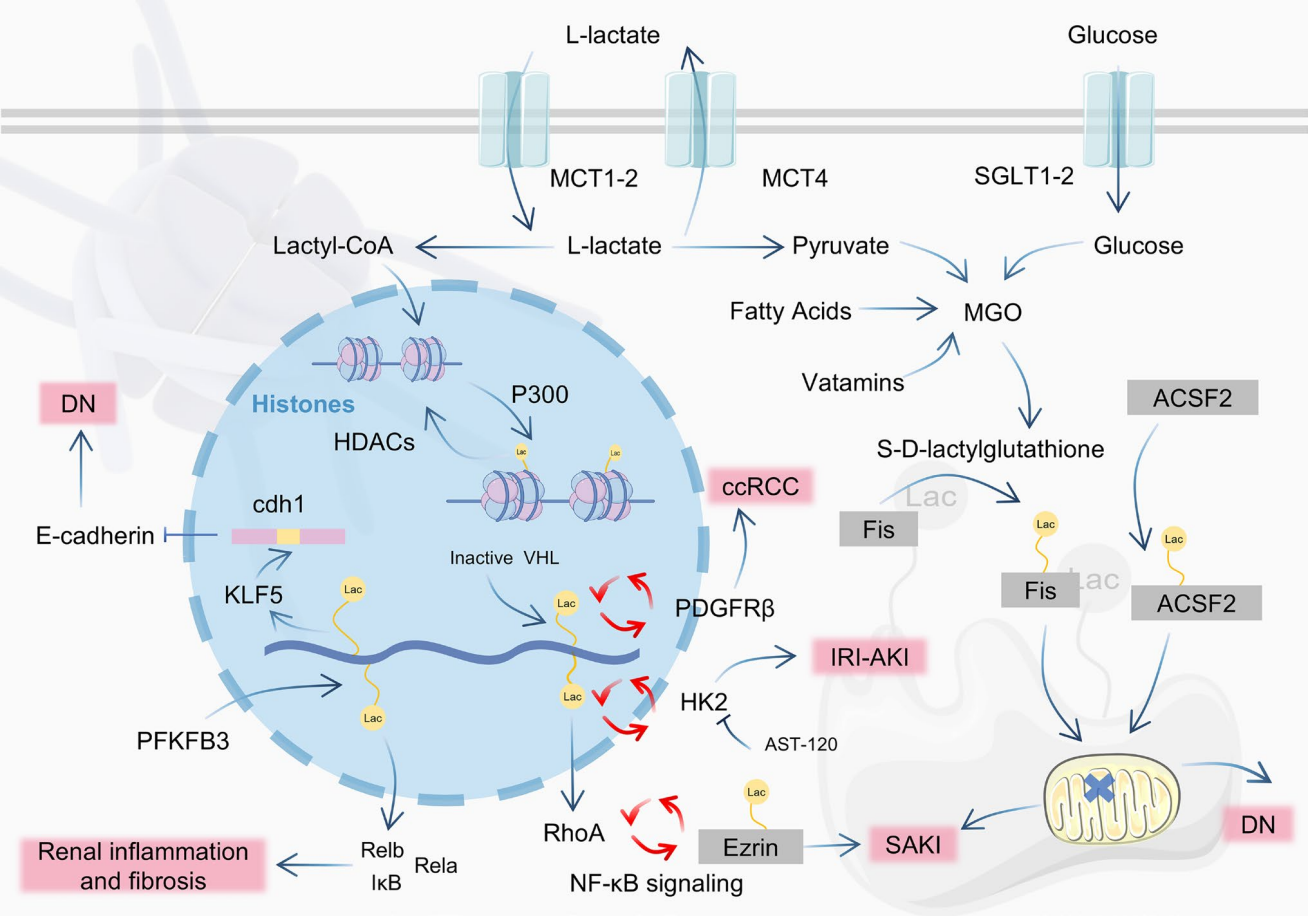
Lactylation in kidney diseases. Research on histone and nonhistone lactylation in kidney diseases can be categorised into two pathways: Enzymatic and nonenzymatic processes. This study highlights the relationships between lactylation and various inflammatory and fibrotic regulatory pathways in kidney diseases, such as acute kidney injury (AKI), diabetic nephropathy (DN) and clear cell renal cell carcinoma (ccRCC). The red circles in the figure illustrate the positive feedback loops associated with lactylation.

Histone lactylation has undergone more extensive research. Studies have focused primarily on several key targets, including H3K18, histone H3 lysine 9 (H3K9), H4K12, histone H3 lysine 14 (H3K14) and histone H4 lysine 5 (H4K5). Qiao et al. reported that H3K18la activates the RhoA‐ROCK1‐Ezrin signalling pathway, increasing ezrin K263 lactylation. This activation subsequently triggers the NF‐κB pathway, leading to inflammatory responses, cell apoptosis and deterioration of kidney function. Glucose transporter 1 (GLUT1) inhibition and the ezrin K263 site mutation can alleviate renal dysfunction [16]. In PTECs, hexokinase 2 (HK2) knockout inhibits hypoxia/reperfusion (H/R)‐induced glycolysis and H3K18la. H3K18la is enriched at the HK2 promoter, leading to an increase in HK2 levels [128]. In clinical and mouse models of CKD, 6‐phosphofructo‐2‐kinase/fructose‐2,6‐biphosphatase 3 is upregulated, resulting in H4K12la. This modification can increase the transcription of NF‐κB signalling pathway‐related genes in PTECs, contributing to renal inflammation and fibrosis. The H4K12la level is positively correlated with the degree of renal inflammation and fibrosis in patients with CKD [17]. In DN, increased H3K14 lactylation (H3K14la) promotes the transcription of Krüppel‐like factor 5 (KLF5), which recognises the cdh1 promoter and inhibits E‐cadherin expression, thereby accelerating the DN progression (Figure 4) [19].

The NF‐κB pathway is involved in the progression of various kidney diseases [28, 29, 129, 130, 131, 132]. The regulatory effects of lactylation on this pathway have been validated in multiple contexts, including cancer, myocardial infarction (MI), immune‐related inflammation and chemotherapy resistance. Furthermore, the RhoA‐ROCK1 pathway contributes to AKI pathogenesis and renal fibrosis related to CKD [133]. Macrophages play a crucial role in AKI to CKD. M1 macrophages release proinflammatory factors early in AKI, exacerbating renal injury. Conversely, M2 macrophages regulate inflammation, promote cell proliferation and facilitate repair during the later stages of AKI, contributing to AKI to CKD [134]. Although these lactylation‐modified pathways and cells are involved in AKI to CKD, the extent to which PTM mechanisms influence the disease remains unclear, necessitating further investigation.

### 6.2 Effects of Nonhistone Lactylation in the AKI‐to‐CKD Progression

Nonhistone lactylation is also involved in the AKI‐to‐CKD pathogenesis. An et al. demonstrated that hyperacetylation or inactivation of pyruvate dehydrogenase E1 component subunit alpha mediates the lactylation of mitochondrial fission protein 1 (Fis1). Fis1 K20 lactylation (Fis1 K20la) exacerbates excessive mitochondrial division by interacting with dynamin‐related protein 1 (Drp1), worsening SAKI. Lactate production inhibition and reduced Fis1 K20la were demonstrated to alleviate the SAKI severity [15]. In 2024, a study published in Diabetologia reported significantly elevated lactylation levels in the kidneys of patients with diabetes and diabetic db/db mice. A total of 356 lactylation sites were identified in 165 proteins, most of which are located in the mitochondria and play crucial roles in mitochondrial metabolism. Specifically, acyl‐CoA synthetase family member 2 (ACSF2) lysine 182 lactylation (K182la) was revealed to cause mitochondrial dysfunction, promoting disease progression in DN (Figure 4) [18].

During the progression from AKI to CKD, nonhistone lactylation mediates mitochondrial dysfunction, exacerbating renal damage. However, the diversity of nonhistone proteins and their modification sites presents significant research challenges. One potential strategy might involve initial data‐driven screening to identify relevant targets, followed by experimental validation. Alternatively, focusing on nonhistone proteins that are closely related to AKI‐to‐CKD metabolic mechanisms could be pursued. This approach, which is commonly adopted in studying nonhistone lactylation in other diseases, could offer a useful reference for investigation.

## 7 The Role of Lactylation in Other Kidney Diseases

In addition to the established mechanisms, numerous proteins regulating lactate in kidney diseases have also been reported to regulate lactylation in other kidney diseases (Table 6). A study conducted in patients with ccRCC revealed that Von Hippel–Lindau (VHL) inactivation triggers histone lactylation, activating PDGFRβ transcription. This activation stimulates histone lactylation, creating a positive feedback loop that accelerates the ccRCC progression [111]. PDGFRβ amplification and activation in renal mesenchymal cells lead to the pathological proliferation of mesangial cells and interstitial fibroblasts, which differentiate into myofibroblasts. This process causes mesangial sclerosis and interstitial fibrosis, manifesting as a reduced glomerular filtration rate and renal anaemia [135, 136]. Whether the stimulatory effect of PDGFRβ signalling on histone lactylation plays similar regulatory roles in renal fibrosis and CKD warrants further investigation.

The zinc finger transcription factor, GLIS family zinc finger 1 (Glis1), initiates the glycolytic gene expression in the early stages of cellular reprogramming, elevating lactate and acetyl‐CoA levels. This elevation promotes the acetylation (H3K27Ac) and lactylation (H3K18la) levels, facilitating cellular reprogramming [137]. Glis1 is a protective gene that significantly influences cellular ageing and renal fibrosis by maintaining mitochondrial stability and cellular integrity, thereby delaying age‐related renal fibrosis progression [138, 139]. Further studies are necessary to determine whether the protective mechanism of Glis1 in renal fibrosis is associated with lactylation.

In addition, as key delactylation enzymes, HDACs play a protective role in kidney function and are essential for renal regeneration following AKI [140]. Other studies have indicated that aristolochic acid (AA) can upregulate the expression of HDACs in renal tissues, contributing to AKI development. Inhibition of HDACs can promote the recovery of renal function and counteract renal fibrosis induced by AA [141]. The relationship between the regulation of HDACs and lactylation in AKI development warrants further exploration.

## 8 Outlook

As research progresses, lactate has been increasingly recognised for its multifaceted roles as an energy source, gluconeogenic precursor, signal transduction molecule and protein modification group. While previous studies have primarily explored the first three functions of lactate, studies on lactylation are still emerging. Research on lactylation has mainly focused on cancer, inflammatory diseases, ischaemic–hypoxic injuries and neurodevelopment. Glycolysis and lactate signalling are prominent in both healthy and diseased kidneys, highlighting the distinct and crucial role of lactate metabolism in the kidneys. Generally, lactate has been used as a biomarker for disease state, treatment efficacy, and prognosis in critical illnesses, but few studies have explored how it may affect disease progression at the molecular level.

This review summarises the research advancements in lactate and lactylation in the AKI‐to‐CKD progression. During the AKI progression, elevated lactate levels predominantly have a detrimental effect on the kidneys and promote disease progression. The mechanism of renal damage is frequently associated with mitochondrial dysfunction. Lactate can interfere with cellular death pathways such as autophagy, apoptosis and senescence and induce immunosuppression. However, some studies have revealed that lactate may exert a nephroprotective effect by inhibiting inflammation. In CKD models, including PKD, DN, and hypertensive nephropathy, lactate accumulation consistently exacerbates renal fibrosis and impairs renal function. Studies on AKI‐to‐CKD have demonstrated that lactate promotes the transdifferentiation of perirenal cells, thereby facilitating renal interstitial fibrosis and progression from AKI to CKD.

As a novel PTM, lactylation involves the covalent attachment of lactyl groups to proteins. It plays a crucial role in cellular metabolism, gene expression and signal transduction, suggesting new directions for research on various diseases. Although studies on lactylation are still in their infancy, their regulatory effects are already recognised as extensive and complex, influencing different organs, multiple systems and various pathological processes. This widespread presence supports the notion that lactate plays a broad regulatory role across multiple diseases. Most studies have focused predominantly on cancer, especially in the gastrointestinal tract, liver, brain and lungs. Other well‐studied areas include neurodevelopmental disorders, organ fibrosis, immune‐related inflammation and ischemic–hypoxic injuries.

Most studies indicate that lactylation promotes disease progression, although a few have suggested a protective role, reflecting the complex regulatory effects of lactate. Notably, research on lactylation in kidney diseases remains limited compared to that in cancer. Only a few studies focused on AKI‐to‐CKD have demonstrated the presence of lactylation, suggesting that both histone and nonhistone lactylation contribute to kidney disease development and play significant roles. While evidence indicates that lactylation may contribute to renal damage, the precise mechanisms involved in the AKI‐to‐CKD progression remain unclear, and further research is needed to explore its potential.

Although some studies have established the role of lactylation in the AKI to CKD progression, many critical questions remain unresolved. Current perspectives on lactylation mechanisms suggest that histone lactylation is predominantly mediated by specific enzymatic processes, whereas nonhistone lactylation occurs mainly through nonenzymatic processes. However, these viewpoints are based on limited research and remain speculative. The specific roles of l‐lactate and d‐lactate in contributing to lactylation and whether these forms of lactate modify proteins differently in terms of function remain unclear. Furthermore, how nonspecific histone lactylation enzymes selectively catalyse acyl modifications and whether cross‐talk with other acylation processes influences the regulation of physiological states has yet to be elucidated. Regarding the study of AKI to CKD, it is unclear whether lactylation plays a substantial role in disease regulation or holds significant clinical relevance. These uncertainties highlight the need for further investigations to verify the impact of lactylation.

This review provides an overview of the key concepts related to lactate, outlines the metabolic processes of lactate in both healthy and diseased kidneys and reevaluates the critical role of lactate in kidney diseases. This review also discusses the specific mechanisms by which lactate and lactylation regulate the progression from AKI to CKD, alongside an analysis of the current state of lactylation research. The goal is to provide insights and references for future research, encouraging further investigations into the role of lactate and lactylation in AKI to CKD. Considering the crucial role of lactate and lactylation in AKI to CKD, targeting the regulation of both lactate and lactylation may become a viable therapeutic strategy.

## Author Contributions

Yi Hou, Qi Feng and Fengxun Liu conceptualised the ideas and wrote the manuscript. Yi Hou, Dongwei Liu, Zuishuang Guo, Cien Wei, Fengyu Cao, Yue Xu, Qi Feng and Fengxun Liu reviewed and revised the manuscript. All the authors have read and approved the final version of the manuscript being submitted.

## Conflicts of Interest

The authors declare no conflicts of interest.

## Data Availability

All data supporting this study are available in the published literature, as cited in the manuscript.
